# Retention in primary care among unstably housed residents of a low-income, inner-city neighborhood with a high prevalence of substance use and related disorders

**DOI:** 10.1186/s12939-024-02332-y

**Published:** 2024-11-28

**Authors:** M. Gumprich, W. Zhang, J. Li, K. Salters, R. Barrios, P. Sereda, C. Stanley, R. Joe, D. Hall, V. Lima, G. Sincraian, A. Marante Changir, R. Parry, C. Fulton, T. Wesseling, J. Montaner, S. Parashar, David M. Moore

**Affiliations:** 1 BC Centre for Excellence in HIV/AIDS, Vancouver, Canada; 2 Faculty of Medicine, University of British Columbia, Vancouver, Canada

**Keywords:** Primary healthcare, Retention in care, Substance use disorders, Mental health, Homelessness, Unstable housing

## Abstract

**Introduction:**

Access to and engagement with primary healthcare can be difficult for marginalized low-income populations residing in inner cities in high-income countries. We designed a study to examine retention in primary care among clients of a novel interdisciplinary primary care clinic in the Downtown Eastside of Vancouver, Canada who did not previously have access to care.

**Methods:**

Beginning in June 2021, clients of the Hope to Health clinic were offered enrolment in a cohort study which involved a baseline and follow-up surveys every six months, and linking their data to information from the clinic’s electronic medical records. We used Chi-square or Fisher’s Exact test and Wilcoxon rank sum test to compare clients who were lost to follow-up (LTFU) or deceased, with clients who were retained in care at the end of follow-up, Cox proportional hazards modeling was used to examine independent associations with mortality or LTFU.

**Results:**

Among 425 participants enrolled, the median age was 50 years (IQR 40–59), 286 (67.3%) participants were men and 128 (25.4%) were unstably housed at enrollment. Among 338 participants with at least six months of follow-up after enrolment, 262 participants (67.5%) were retained in care, 20 (5.2%) had moved, 57 (14.7%) were classified as LTFU, and 28 (7.2%) had died with a median of 19.9 months of follow-up time. The risk of death or LTFU was independently associated diagnosed with alcohol use disorder (AUD) (adjusted hazard ratio [AHR] = 2.23 vs. not; 1.38–3.60), frequency of medical doctor visits (AHR = 0.69 per visit per 3 months; 0.60–0.79) and social work visits (AHR = 0.73 per visit per 3 months; 0.59–0.90. Stimulant use disorder or asthma were not significantly associated with retention in care.

**Conclusion:**

We found that a primary healthcare model of care was successful in retaining over two-thirds of clients in primary healthcare after more than 18 months of follow-up. Additional supports for those diagnosed with alcohol use disorder are needed to retain them in care.

## Introduction

Access to and engagement with primary healthcare (PHC) can be difficult for people living with substance use disorders, unstable housing, mental and/or physical health conditions, low-to-no income, or any combination of the aforementioned. Within this population, it is known that those most in need of PHC are also those less likely to receive this care [1]. Unstable housing is associated with more frequent emergency department (ED) visits and hospital use in comparison to those who are housed [2–4]. A 2014 study looking at PHC usage by those who are homeless in high-income countries found that those who are unhoused can frequent the ED more than three to five times a year, which is much higher than the general population [4]. Common barriers to accessing PHC instead of ED are: not having all needed health and social supports in one place, mental health issues, multiple comorbidities, lack of financial security, poor neighborhood conditions [5, 6], and fear and lack of trust in the healthcare system [7].

People who are unstably housed or have low socioeconomic status are more likely to engage with and return to PHC settings that are tailored to their needs [6, 8, 9]. Once this population is engaged with tailored PHC, they turn to their health care team for advice and help rather than the hospital [10]. The strengthened patient-provider relationship also increases the likelihood of disclosing or identifying health issues that would have been otherwise undetected [6]. Connection is key at tailored clinics, as those who are economically disadvantaged, have a mental illness, and/or a substance use disorder have had success in accessing PHC services when supported by a “care manager,” meaning someone who helps advocate for the patient and helps the patient communicate with care providers [11]. Previous research regarding inner-city populations and PHC has mainly investigated those who are unstably housed, but has largely overlooked the effects of the combination of multiple comorbidities, substance use, and mental health disorders in terms of their influence on accessing primary care [4, 8] and what services this population wants to have at a tailored clinic [6, 12].

The Downtown Eastside (DTES) of Vancouver, British Columbia, has the largest population of those who are homeless in Greater Vancouver, and many residents have complex medical and social needs [13]. Homelessness also appears to be increasing in Vancouver. As of 2023, there was an increase of 16% compared to 2020, with 2,420 homeless people [13]. Among the homeless counted in 2020 and 2023, 63% had two or more medical conditions [13, 14]. In addition, Vancouver is in the midst of a toxic drug crisis, with the DTES having a 10 times greater overdose death rate than the provincial average [15].

Residents of the DTES have high rates of ED visits and hospitalizations, showing the need for tailored PHC services for this community [13]. 53% (1,260 people) of the homeless population surveyed in 2023 used an ED in the previous year, 46% used non-emergent hospital services, and 42% used an ambulance [13]. An estimated 6000 to7,000 residents in the DTES are unattached to PHC [16], meaning they do not have a regular primary care provider. In response to this problem, the BC Centre for Excellence in HIV/AIDS, Vancouver Coastal Health, and the BC Ministry of Health developed a new interdisciplinary PHC clinic known as The Hope to Health Research and Innovation Complex (H2H), which opened in September 2019 [17]. H2H was designed to provide interdisciplinary PHC for people living in the DTES of Vancouver who did not previously have access to care.

### Objectives

We designed an analysis to examine retention in PHC over time among a sample of H2H clients enrolled in a cohort study. Furthermore, we examined how experiences of unstable housing, demographic characteristics, specific medical conditions, and services affected the likelihood of being retained in care.

## Methods

### Setting and study design

Study participants were primary care clients of the H2H Centre. H2H has a supervised consumption site, a drug testing site, a safer drug supply program, and a collocated primary care clinic. H2H clients receive access to harm reduction, primary health care and support related to housing, income, behavior, and social needs. Clients are assigned to a primary care team comprised of a family doctor, social worker, peer support worker, and nurses. These teams are supported by an on-site clinical pharmacist, behavioral consultant, and phlebotomist [17]. All patients are case-managed, meaning the care team periodically reviews them to assess engagement in care and progress in achieving care standards or goals. At H2H, there is dedicated protected time and leadership support for the team to focus on key quality outcomes and do targeted quality improvement work.

The clinic offers both booked appointments and walk-in clinic visits. At clinic enrollment, clients are asked about the best means to contact them and the clinic provides text message or phone call reminders for upcoming appointments based on these preferences where possible. Peer outreach workers can also assist clients to attend appointments.

### Subjects

We launched the HERE (Hope to Health Engagement and Retention Evaluation) Study and beginning in June 2021 enrolled H2H clients who: (i) were 19 years of age or older; (ii) were able and willing to provide informed consent to study procedures; (iii) were residing or accessing regular healthcare services in the DTES; (iv) self-identified as requiring PHC for one or more chronic conditions; (v) were not currently engaged in PHC services at another service provider; (vi) had attended an intake visit with a nurse or social worker, and had at least one visit with a H2H physician; and (vii) were able to understand and complete enrollment procedures in English.

The study was advertised to clients in the H2H Centre. Peer support workers and medical office assistants provided H2H clients with study information while they were waiting to see service providers. When a client expressed interest in the study, a Peer Research Associate (PRA) - a trained interviewer with similar lived experience to the patient population - scheduled an appointment to review the study procedures and inclusion criteria with the client. If the client was interested, the PRA completed informed consent procedures with the participant and administered the enrolment survey. As part of consent procedures, participants provided authorization to link questionnaire data to the electronic medical records (EMR) of the H2H clinic as well as to the BC Vital Statistics registry, which records all deaths in the province. All participants provided informed consent.

Participants completed a required baseline and follow-up surveys administered by a PRA every six months after study enrollment for a maximum of three years. The surveys were conducted in person, with questions and answer options being read to clients by PRAs. Baseline surveys took 30–60 min to complete, and follow-up surveys took 30 min on average. All participants received an honorarium of $40 for each study visit. Participants were reminded of their study appointments, or that they were due for a follow-up survey through various methods, including phone calls, email, text messaging, mail, in person in the clinic lobby, or another mutually agreed-upon method. Contact was attempted three times for participants who missed a survey appointment. However, participants were able to continue in the study even if they missed survey appointments and reappeared at a later date. The study received scientific and ethics approval from the University of British Columbia Research Ethics Board at the St. Paul’s Hospital site (REB number H20-03256).

### Data collection

The survey included questions on demographics, including sex assigned at birth and gender identity, social supports, ethnicity, and income. We asked all participants “In your lifetime, have you ever been homeless?”, with response options of “ Yes, currently I am homeless”; “Yes, I have been homeless in the past 3 months but not now”; “Yes, I have been homeless in the past but more than 3 months ago” and “No, I have never been homeless” The question also included the following definition of homelessness: “For this survey homelessness means living on the street, in an emergency shelter, in interim (or transitional) housing, living temporarily with others (couch surfing), living in institutional contexts (hospital, prison), or without permanent housing arrangements.” We also asked about previous and current experiences with PHC, current use of ancillary health services (e.g., substance use treatment), and their top three health concerns. We enquired about recent substance use using the ASSIST scale [18], and participants were systematically surveyed for symptoms of anxiety using the Generalized Anxiety Disorder 7-item (GAD-7) [19] and depression using the Centre for Epidemiologic Studies Depression Scale 10-item version (CESD-10) [20].

The survey also collected information on, health care needs, service utilization, and health-related behaviors. Clinical and health service-related outcomes were captured through the EMR and linked to the survey responses. Our primary outcome measure was death or loss-to-follow-up (LTFU), defined as not having at least one documented physician visit in the EMR in any three-month period and no subsequent visit. Follow-up continued until June 30, 2023.

### Data analysis

We calculated descriptive statistics for all outcomes and explanatory variables. Explanatory variables were categorized into sociodemographics, medical diagnoses, and health service variables (physician and social worker visits). All medical conditions were physician-diagnosed. Anxiety and depression scores higher than 10 were considered to be significant symptoms. Health services visits were based on EMR data.

We used Chi-square or Fisher’s Exact test for categorical variables, and Wilcoxon rank sum test for continuous variables to compare clients who were LTFU or deceased, with clients who were retained in care. Data were censored for participants who transferred out of the clinic, moved out of the clinic catchment area, or withdrew from the clinic on the date of their last clinic service. Univariable and multivariable Cox proportional hazards models were used to examine factors associated with time to death, or LTFU, from enrolment among study participants with at least six months of follow-up from study enrollment to June 30, 2023. Variables associated with the outcome in univariable analysis at a significance < 0.05 were considered for inclusion in the multivariable model. Variables for inclusion in the final model were based on the minimization of the Akaike information criterion (AIC) and type III *p*-values [21]. All analyses were conducted in SAS version 9.4 (Cary, North Carolina, USA).

## Results

As of June 30, 2023, 425 participants had enrolled in the HERE Study. The median age was 50 years (IQR 40–59). Two hundred eighty-six (67.3%) participants were men, 132 (31.1%) were women, and seven (1.7%) identified as transgender or other gender nonconforming identities. A total of 234 (55.1%) identified as Caucasian or White, 140 (32.9%) identified as Indigenous, 12 (2.8%) as Black, African or Caribbean and 11 (2.6%) as Latin, Central or South American.

One hundred thirty-three (31.3%) participants reported an annual income less than $10,000 and 245 (57.7%) had an income of $10,000-$19,999. One hundred eight (25.4%) reported being homeless at enrollment, 102 (24.0%) had been homeless in the three months prior to enrollment, and 162 (38.1%) had been homeless more than three months prior. Three hundred forty-seven (81.7%) participants reported that they did not have a sufficient amount of food in the last year. The most commonly reported top three health concerns were mental health (95, 22.4%), drug use or addiction (91, 21.4%) and chronic pain (38, 9.0%).

A total of 368 participants (86.6%) reported having ever used psychoactive substances. The most commonly reported substances used in the three months prior to enrollment were tobacco (321, 75.5% reporting current use), opioids (221, 52.0%), cannabis (194, 45.7%), amphetamine-type stimulants (175, 41.1%) and alcohol (170, 40.0%). Of those reporting opioid-use in the past three months, 68.3% reported taking medications to treat opioid use disorder (OUD, inclusive of methadone, slow-release oral morphine, or buprenorphine).

Table 1 provides details on the 388 (91.3%) participants who had at least six months of follow-up time after study enrolment who were included in the analysis of retention in care. Among these, 262 participants (67.5%) were retained in care, 20 (5.2%) had moved, 57 (14.7%) were classified as LTFU, and 28 (7.2%) had died in a median of 19.9 months of follow-up time. There were no significant differences between participants retained in care and those who were not in terms of age, gender, experience with homelessness, or food security. However, participants who were LTFU or deceased had fewer medical doctor (MD) and social worker (SW) visits (median of 2 and 0.5 per 3-month period, respectively) at H2H compared to those still engaged in care (medians of 4 and 0.7 per 3-month period, respectively; *p* < 0.001 for MD visits and 0.045 for SW visits) (Table 1). Participants living with OUD or stimulant use disorder (St UD) were more likely to be retained in care (80.9% and 82.5%, respectively; *p* < 0.001 for both) than those without these disorders (63.3% and 67.0% respectively; *p* < 0.01 for both). Conversely, participants living with an alcohol use disorder (AUD) were less likely to be retained in care (64.9%) than those without (76.4%; *p* = 0.044). There were no differences in the distribution of homelessness experience and other comorbidities (cardiovascular disease, COPD, diabetes, HIV, hepatitis C, asthma, hypertension, chronic liver disease, chronic pain, ADHD, and schizophrenia or other psychosis) comparing those who became LFTU or who died with those who were retained in care.

**Table 1:** Bivariate analysis of factors associated with LTFU or mortality among HERE study participants

Explanatory variables	Total *n* (column %)	Retained in care *n* (row %)	Lost to follow up or Deceased *n* (row %)	*P*-value
**Demographics**				
**Gender**				
Female	112 (31.6)	91 (81.3)	21 (18.8)	0.068
Male	240 (67.8)	169 (70.4)	71 (29.6)	
Other	2 (0.6)	2 (100)	0	
**Age**				
Median years (Q1-Q3)	50 (40–59)	49 (41–58)	51 (40–60)	0.523
**Homelessness experience**				
Currently homeless	86 (24.3)	67 (77.9)	19 (22.1)	0.281
Homeless < 3 months ago but not currently	88 (24.9)	61 (69.3)	27 (30.7)	
Homeless > 3 months ago	135 (38.1)	104 (77.0)	31 (23.0)	
Never been homeless	44 (12.4)	29 (65.9)	15 (34.1)	
**Total annual income**				
< $10,000	117 (33.1)	84 (71.8)	33 (28.2)	0.950
$10,000 - $19,999	200 (56.5)	148 (74)	52 (26)	
$20,000 - $29,999	18 (5.1)	14 (77.8)	4 (22.2)	
≥ $30,000	5 (1.4)	4 (80)	1 (20)	
**Food security**				
Not sufficient	294 (83.1)	217 (73.8)	77 (26.2)	0.570
Sufficient	45 (12.7)	35 (77.8)	10 (22.2)	
**Physician diagnosed medical conditions**				
**Opioid use disorder**				
No	139 (39.3)	88 (63.3)	51 (36.7)	< 0.001
Yes	215 (60.7)	174 (80.9)	41 (19.1)	
**Stimulant use disorder**				
No	194 (54.8)	130 (67.0)	64 (33.0)	< 0.001
Yes	160 (45.2)	132 (82.5)	28 (17.5)	
**Alcohol use disorder**				
No	280 (79.1)	214 (76.4)	66 (23.6)	0.044
Yes	74 (20.9)	48 (64.9)	26 (35.1)	
**Anxiety**				
No	262 (74.0)	191 (72.9)	71 (27.1)	0.421
Yes	92 (26.0)	71 (77.2)	21 (22.8)	
**Depression**				
No	278 (78.5)	203 (73.0)	75 (27.0)	0.417
Yes	76 (21.5)	59 (77.6)	17 (22.4)	
**Cardiovascular disease**				
No	270 (76.3)	198 (73.3)	72 (26.7)	0.602
Yes	84 (23.7)	64 (76.2)	20 (23.8)	
**Hepatitis C**				
No	205 (57.9)	145 (70.7)	60 (29.3)	0.099
Yes	149 (42.1)	117 (78.5)	32 (21.5)	
**Asthma**				
No	323 (91.2)	235 (72.8)	88 (27.2)	0.082
Yes	31 (8.8)	27 (87.1)	4 (12.9)	
**Anxiety and depression scores at enrollment**				
**GAD-7 Anxiety score**				
< 10 (Minimal to mild)	206 (58.2)	147 (71.4)	59 (28.6)	0.178
≥ 10 (Moderate to severe)	144 (40.7)	112 (77.8)	32 (22.2)	
**CES-D depression score**				
< 10	230 (65.0)	70 (65.4)	37 (34.6)	0.026
≥ 10 (Significant symptoms)	107 (30.2)	177 (77.0)	53 (23.0)	
**Health services visits**				
**Medical doctor visits**				
# per 3 months - Median (Q1-Q3)		4 (3–5)	2 (1–3)	< 0.001
**Social worker visits**				
# per 3 months - Median (Q1-Q3)		0.7 (0.2–1.7)	0.5 (0.1–1.3)	0.045

 Table 2 examines the impact on specific medical diagnoses on the rate of both MD and SW visits per three months. Participants with OUD had more frequent MD visits (median of 3.9 visits for those with OUD vs. 2.5 without; *p* < 0.001) but less frequent SW visits (median of 0.5 visits for those with OUD vs. 0.9 without; *p* = 0.004). Participants with St UD had more frequent MD visits (median of 3.7 visits for those with St UD vs. 3.0 without; *p* = 0.008) but no difference in SW visits (median of 0.5 visits for those with St UD vs. 0.7 without; *p* = 0.364). Diagnoses of depression or AUD had no impact on MD visits, but participants with those conditions did have more frequent SW visits compared to those who did not (*p* = 0.006 for depression and *p* < 0.001 for AUD).

**Table 2:** Medical and social work visits per three months by diagnosed substance use disorder amongst HERE Study participants

Diagnosis	Median number of visits	Total n (%)	P-value
**Medical doctor visits**			
**Stimulant use disorder**			
No	3.0	215 (55.6)	0.008
Yes	3.7	172 (44.4)	
**Alcohol use disorder**			
No	3.4	306 (79.1)	0.328
Yes	3.1	81 (20.9)	
**Opioid use disorder**			
No	2.5	155 (40.1)	< 0.001
Yes	3.9	232 (59.9)	
**Depression**			
No	3.3	308 (79.6)	0.319
Yes	3.5	79 (20.4)	
**Social worker visits**			
**Stimulant use disorder**			
No	0.7	215 (55.6)	0.364
Yes	0.5	172 (44.4)	
**Alcohol use disorder**			
No	0.5	306 (79.1)	< 0.001
Yes	1.2	81 (20.9)	
**Opioid use disorder**			
No	0.9	155 (40.1)	0.004
Yes	0.5	232 (59.9)	
**Depression**			
No	0.5	308 (79.6)	0.006
Yes	0.9	79 (20.4)	

In our final multivariable Cox proportional hazards model (Table 3), we found that the risk of death or LTFU was higher among those living with an AUD (adjusted hazard ratio [AHR] = 2.23; 1.38–3.60). In terms of health services, we found that both MD visits (AHR = 0.69; 0.60–0.79) and social work visits (AHR = 0.73; 0.59–0.90) were associated with a lower hazard of death or LTFU. Notably, experiencing homelessness was not associated with LTFU or death in the univariable analysis and was not included in our final model. Despite being associated with the outcome in the univariable analysis, OUD diagnosis was not selected for inclusion in our multivariate model. As seen in Table 2, OUD was associated with much higher rates of MD visits and therefore appeared to be co-linear with this variable. We ran an additional multivariable model where OUD replaced MD visits in the model and its association was retained (AHR = 0.50; 95% CI 0.32–0.79). However, the overall AIC was higher in this model indicating that it was not a better fit for our data.

**Table 3:** Cox proportional hazards analysis of variables associated with LTFU or death among HERE study participants

Explanatory variables	Unadjusted hazard ratio	*P*-value	Adjusted hazard ratio	*P*-value
**Demographics**				
**Age** (per 10-year increment)	1.02 (0.86–1.20)	0.844		
**Gender**				
Female	1.00			
Male	1.44 (0.88–2.34)	0.146		
**Homelessness Experience**				
Never been homeless	1.00			
Currently homeless	0.60 (0.30–1.18)	0.137		
Homeless < 3 months ago but not currently	0.76 (0.41–1.43)	0.398		
Homeless > 3 months ago	0.63 (0.34–1.17)	0.141		
**Total annual income**				
< $10,000	1.00			
≥ $10,000	0.80 (0.52–1.23)	0.300		
**Food Security**				
Not sufficient	1.00			
Sufficient	1.02 (0.53–1.97)	0.963		
**Physician diagnosed medical conditions**				
**Opioid use disorder**				
No	1.00			
Yes	0.52 (0.35–0.79)	0.002		
**Stimulant use disorder**				
No	1.00		1.00	
Yes	0.53 (0.34–0.83)	0.006	0.68 (0.43–1.07)	0.091
**Alcohol use disorder**				
No	1.00		1.00	
Yes	1.69 (1.07–2.67)	0.024	2.23 (1.38–3.60)	0.001
**Anxiety**				
No	1.00			
Yes	0.89 (0.54–1.45)	0.632		
**Depression**				
No	1.00			
Yes	0.69 (0.41–1.17)	0.170		
**Cardiovascular disease**				
No	1.00			
Yes	0.64 (0.39–1.06)	0.085		
**Hepatitis C**				
No	1.00			
Yes	0.73 (0.48–1.13)	0.157		
**Asthma**				
No	1.00		1.00	
Yes	0.37 (0.14-1.00)	0.051	0.36 (0.13–1.02)	0.055
**Anxiety and depression scores**				
**GAD-7 Anxiety score**				
< 10 (Minimal to mild)	1.00			
≥ 10 (Moderate to severe)	0.86 (0.56–1.32)	0.490		
**CES-D Depression score**				
< 10	1.00			
≥ 10 (Significant symptoms)	0.69 (0.10–1.66)	0.078		
**Health Services Visits**				
**Medical doctor visits**				
# per 3 months	0.64 (0.56–0.73)	< 0.001	0.69 (0.60–0.79)	< 0.001
**Social Worker visits**				
# per 3 months	0.70 (0.58–0.86)	< 0.001	0.73 (0.59–0.90)	0.003

## Discussion

In our analysis of low-income unstably housed people with a high prevalence of substance-use and related disorders in Vancouver, we found that approximately two-thirds were still retained in care after over 18 months of follow-up time. Of those who enrolled in the study, 7% had died and another 5% had moved, leaving only 14% who were truly lost to follow-up. Considering the challenges faced by this population, this indicates excellent success in retaining clients in PHC. The H2H model, in which clients can receive nearly all of their needed supports (medical, social, and psychological) is working well to ensure people are sustainably engaging with their needed PHC. This model can be replicated by other inner-city clinics to serve their community.

Among the factors we found associated with a reduced risk for mortality or LTFU, two were related to services offered by the clinic. Participants with more frequent physician visits and social work visits were less likely to be LFTU or die. These findings are similar to previous studies showing that engagement with medical care at tailored clinics increases one’s usage of PHC [8, 10]. Having tailored services provides the opportunity to address a client’s socioeconomic needs alongside their medical needs, providing a holistic approach to care.

Those with an OUD and St UD were more likely to be retained in care, in our bivariate analysis and the univariable Cox model analyses. although not in our final multivariable model. This suggests that these diagnoses were not independently associated with our outcome but are explained by other factors in our multivariable model. This was likely due to some collinearity of MD visits and both OUD and St UD, where participants with each of these conditions had much higher frequency of visits than those without. Again, given the impact of the opioid overdose crisis in British Columbia, where the DTES is an epicentre, this demonstrates that H2H is appropriately meeting the needs of this population. The clinic provides a broad range of services for OUD ranging from supervised consumption, distribution of harm reduction supplies, provision of novel opioid substitution therapies such as injectable diacetylmorphine and hydromorphone and fentanyl patches, prescribed hydromorphone tablets as a safer alternative to street opioids, as well as more conventional opioid agonist therapies such as buprenorphine, methadone, and sustained-release oral morphine. H2H offers active follow-up of all clients and provides bridging scripts to maximize positive outcomes of treatment. The clinic also provides referrals to other local agencies which provide detoxification services as well as residential treatment programs. Given the relative lack of success in finding medications to help with St UD, the clinic has also developed a program for those with a St UD, which combines two psychological interventions, contingency management and cognitive behavioural therapy [22]. Through targeted changes like the introduction of “disengagement rounds”, text appointment reminders, and modifications to the first visit checklist, the team has improved our quality improvement outcome of “disengagement rate”, and this has been sustained over multiple years.

However, those with AUD had a greater risk of LTFU or death than those without this disorder. This high risk of LTFU highlights a need for an increased focus on addressing alcohol use in this population as nearly 20% were diagnosed with AUD. However, only 3.3% of participants ranked their use of alcohol among one of their top three health concerns. This finding likely reflects the social acceptability of alcohol, and the general lack of concern with the health effects associated with alcohol consumption in Canadian society [23], even though the most recent updates on alcohol consumption state that any more than two drinks a week increases one’s risk of a variety of cancers and other health impacts [24]. However, the clinic has already noted the lack of engagement specifically for AUD, and has begun a quality improvement initiative to address this. This has resulted in the introduction of more systematic screening for AUD, offer of medication assisted therapy and referrals to local services for those who are interested in reducing or stopping their alcohol use. The team is also piloting a SMART recovery group onsite.

While previous studies have found housing status to significantly impact PHC access [2], it was not a significant influencing factor for our participants’ engagement with care. This again shows the success of H2H in retaining clients who could most benefit from the services offered. H2H was created with a focus on engaging the most marginalized people in care, and the plan of care is proving to be effective. To this end, the centre is well-staffed with peer support workers who can provide outreach into the community and assist clients in attending appointments, not just at H2H but for specialists, diagnostic tests, and other procedures in Vancouver. This finding does not undermine the importance of access to housing, as one quarter of our participants reported being homeless at the time of study enrolment. However, many clients may have found stable housing as a result of the visits with SWs, which may have reduced the impact of unstable housing over time.

## Limitations

Our study has a number of strengths as well as limitations. Firstly, our study benefitted greatly from linking self-reported survey data to the clinic EMR and BC Vital Statistics, so as to have an objective outcome measure for all participants. From this data linkage, we can accurately account for deaths amongst patients who have not been in the clinic. However, we did not enroll clients from their initial visit at the clinic and as such we may have oversampled individuals who did not become LTFU shortly after engaging with the clinic.

Also, participants in the HERE Study may not be representative of all clients at H2H. In particular, the HERE Study has a much higher proportion of participants with OUD (60.7% vs. 24.5%) and stimulant use disorder (45.2% vs. 17.7%) than the clinic as a whole (R. Barrios and G. Sincraian, personal communication, May 11, 2022). However, the proportion of active clients noted in the clinic EMR (70%) is very similar to the result we found in our study.

As well, our results may over estimate engagement in care, in that clients who are interested in enrolling in a multi-year study may also be more likely to be retained in care. Receiving an honorarium for participating in the study may have also increased clinic engagement. With this in mind, our PRAs made recruitment efforts to enrol and follow those who may not have been strongly connected to the clinic. Lastly, the study was only available to those who were able to conduct the study procedures in English, so for those who are not comfortable in English, this may have been a barrier for participation. However, out of a current active client population of approximately 1100 clients, the clinic estimates that only 5 or fewer clients use interpreters for clinic visits. Therefore we do not think this is a major limitation.

## Conclusion

We found that a PHC model of care designed specifically to address the unique challenges of a low-income inner-city population with high levels of homelessness was successful in retaining over two-thirds of clients in PHC after more than 18 months of follow-up. We also found that the more a participant interacts with doctors and social workers at H2H, the less likely they are to become LTFU or die. Those those with AUD were at a greater risk of LTFU or death. Gender, homelessness experiences, and mental health were not significant factors in engaging with PHC, showing the positive impacts of H2H’s efforts to engage those across many domains of potential marginalization. H2H will use the findings of this study to improve its care, with the goal of lowering the number of deaths and LTFU at the clinic.

## Data Availability

No datasets were generated or analysed during the current study.

## Abbreviations

PHC: Primary healthcare
ED: Emergency department
DTES: Downtown Eastside
H2H: The Hope to Health Research and Innovation Complex
PRA: Peer Research Associate
EMR: Electronic medical records
HERE: Hope to Health Engagement and Retention Evaluation
LTFU: Loss–to–follow–up
OUD: Opioid use disorder
MD: Medical doctor
SW: Social worker
AIC: Akaike information criterion
St UD: Stimulant use disorder
AUD: Alcohol use disorder
AHR: Adjusted hazard ratio
